# Improving Peripheral Reading With Noninvasive Transcranial Electrical Stimulation of Early Visual Areas

**DOI:** 10.1167/tvst.14.9.33

**Published:** 2025-09-24

**Authors:** Andrew E. Silva, Melanie A. Mungalsingh, Louise Raudzus, Benjamin Thompson

**Affiliations:** 1 School of Optometry and Vision Science, University of Waterloo, Waterloo, Ontario, Canada; 2 Department of Psychology, Idaho State University, Pocatello, ID, USA; 3 Aalen University, Optics and Mechatronics, Aalen, Baden-Wuerttemberg, Germany; 4 Centre for Eye and Vision Research, Hong Kong, SAR, People's Republic of China; 5 Liggins Institute, University of Auckland, Auckland, New Zealand

**Keywords:** transcranial random noise stimulation (tRNS), transcranial direct current stimulation (tDCS), transcranial electrical stimulation (tES), peripheral vision, reading

## Abstract

**Purpose:**

Noninvasive transcranial electrical stimulation (tES) of the primary visual cortex can reduce crowding in peripheral vision. We investigated the effect of two tES protocols, visual cortex transcranial direct current stimulation (tDCS) and transcranial random noise stimulation (tRNS), on reading using peripheral vision, a task that is limited by visual crowding.

**Methods:**

A double-blind, placebo-controlled experimental design was used. Forty-one heathy adults with normal visual acuity completed one active and one sham tES session. During each session, English sentences were presented one word at a time 10 degrees below fixation. The proportion of words read correctly was assessed before, during, after and 30 minutes after real or sham tDCS (*n* = 21) or tRNS (*n* = 20).

**Results:**

The tRNS elicited a small but significant performance benefit (*P* = 0.035), whereas tDCS had no effect. Strong within-session learning effects were observed for all conditions (*P* = 0.001).

**Conclusions:**

These results add to a growing body of evidence indicating that noninvasive stimulation of the visual cortex can enhance visual processing and may have applications in vision rehabilitation.

**Translational Relevance:**

Noninvasive brain stimulation may enhance how the brain processes visual input from a diseased eye, complementing standard eye-based treatments of retinal and macular diseases.

## Introduction

In peripheral vision, identifying an object within a cluster is more difficult than identifying an object presented alone, an effect called visual crowding.^1^ Visual crowding is particularly challenging for people with central vision loss, typically caused by macular degeneration, who rely on peripheral vision to recognize objects, faces, and written text.^2^

Strategies for reducing visual crowding often involve the introduction of distinguishing features to the objects composing the crowded visual scene. For example, crowding is reduced when the target and flanking objects have different colors or contrast polarities and when their spatial separation is increased.^3^^–^^6^ However, these crowding reduction strategies do not improve peripheral reading, perhaps because they disrupt word shape.^7^^,^^8^ Other studies have examined whether temporally separating the presentation of letters within a trigram or word improves overall letter or word recognition, again with limited success when using temporal frequencies that are fast enough to enable natural reading.^9^^,^^10^

The neural basis of visual crowding involves a multistage network of early and higher-level regions in the visual cortex. The earliest effects of visual crowding can be observed in V1 with functional magnetic resonance imaging (fMRI), whereas more prominent effects appear in later areas.^11^^,^^12^ More recent work has demonstrated the substantial contribution of parietal regions in crowding for complex objects.^13^^,^^14^ Applying noninvasive transcranial electrical stimulation (tES) to the cortex can reduce the strength of visual crowding without changing the visual characteristics of the stimulus, enabling better processing of cluttered visual scenes.^15^ The effect of tES on visual crowding has been examined in participants with normal vision^16^^–^^18^ as well as in patients with visual dysfunction, including macular degeneration,^19^^,^^20^ and amblyopia.^21^ Common stimulation sites include early visual cortex (Oz, O1, and O2)^17^^–^^19^^,^^21^^,^^22^ and later areas of visual processing (P1 and P2).^16^

Although visual cortex tES has demonstrated some promise for enhancing peripheral reading within a single session,^22^ the relative effect of different tES modalities is unclear. One common tES protocol, known as transcranial direct current stimulation (tDCS), involves affixing electrodes to the head and passing a weak electrical current (1–2 milliampere [mA]) through the scalp and skull, stimulating the underlying neurons. The electrical current enters the body through the anode and exits through the cathode. Cortical excitability is increased under the anodal electrode due to neural depolarization and a reduction in the regional concentration of the inhibitory neurotransmitter GABA.^23^ In contrast, the area beneath the cathodal electrode exhibits reduced cortical excitability. Therefore, stimulation paradigms aiming to increase cortical excitability with tDCS will often place the cathode on a theoretically irrelevant location, such as the cheek.^24^

Although anodal tDCS applied to the visual cortex reduces crowding and lateral inhibition,^16^^,^^18^ transcranial random noise stimulation (tRNS) may be a more effective modulator of cortical excitability.^25^^–^^28^ TRNS uses an alternating current with a frequency that varies randomly, typically within the range of 0.1 hertz (Hz) and 640 Hz to coincide with physiologically measured brain oscillations.^29^ As a result, it is possible to deliver equivalent excitatory stimulation through both electrodes concurrently. Protocols exclusively using higher oscillation frequencies (100–640 Hz), known as high frequency tRNS (hf-tRNS), may influence cortical excitability more than protocols using lower frequencies,^25^^,^^29^ although the optimal frequency range is not yet known.^30^

The method of action for tRNS is currently unclear. One commonly proposed mechanism for the immediate effect of online tRNS is called stochastic resonance, whereby weak subthreshold signals are boosted by a small amount of noise to reach the threshold for detection.^31^ Some previous work has supported this hypothesis, demonstrating enhanced sensitivity for near-threshold visual targets when the visual cortex was stimulated with tRNS at a particular strength and worse performance when other tRNS strengths were used,^32^^,^^33^ although this effect has not always been replicated across sensory modalities.^34^

One recent study examined the effect of visual cortex tDCS on reading in participants with central vision loss due to macular degeneration.^22^ To limit the impact of eye movements, sentences were presented on a computer screen using rapid serial visual presentation (RSVP) in which individual words were presented sequentially at specific presentation speeds and text sizes. A differential effect of tDCS was found whereby English readers exhibited a modest improvement after active tDCS but readers of Chinese exhibited no improvement.^22^

Given that hf-tRNS potentially modulates cortical processing more strongly than tDCS and allows both electrodes to contribute to the desired neuromodulatory effect, it may be particularly effective for enhancing peripheral reading. In the current study, we tested this hypothesis in participants with normal vision who received either hf-tRNS or tDCS in a double-blind study using a within-subject placebo control.

## Materials and Methods

### Participants

Forty-one participants were recruited. Twenty-one participants were administered tDCS (10 men and 11 women, age mean = 21 years, age SD = 4 years). Twenty participants were administered hf-tRNS (6 men and 14 women, age mean = 23, age SD = 3 years). All participants had normal or corrected-to-normal vision and wore their regular spectacles or contact lenses during every session. In addition, all participants were required to exhibit no contraindications for brain stimulation. All research procedures received ethics clearance from the University of Waterloo Office of Research Ethics and adhered to the tenets of the Declaration of Helsinki. All participants provided written informed consent after receiving an explanation of the nature and possible consequences of the study.

### Stimulus and Apparatus

#### Rapid Serial Visual Presentation Task

**Figure 1.**
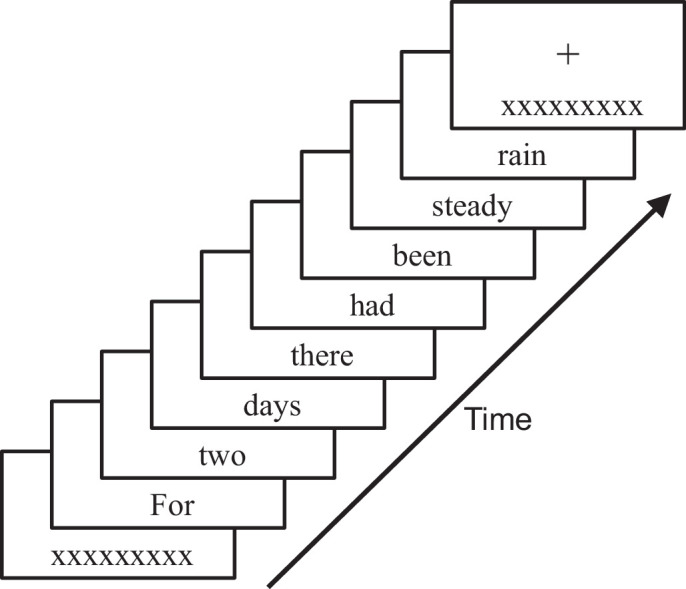
RSVP sentence example. Participants were instructed to fixate on the central cross while a sentence was presented one word at a time 10 degrees below fixation, bounded by “xxxxxxxxx.” The task was to read the sentence aloud, and accuracy was calculated as the total proportion of words read correctly across all presented sentences exhibiting the same presentation speed and text size.

The RSVP test was custom written using the Psychopy library in Python.^35^ The stimulus was presented on an ASUS VG279 27-inch monitor at a pixel resolution of 1920 × 1080 and a refresh rate of 60 Hz. The viewing distance was 65 cm. Participants performed the reading task binocularly. An RSVP trial began by displaying a black fixation cross against a bright background (approximately 150 cd/m^2^). Participants were instructed to fixate on the cross for the full duration of each trial. On every trial, a randomly selected sentence was presented one word at a time 10 degrees below fixation in black Times New Roman font at a particular text size and presentation speed.^36^ In addition, a mask of “xxxxxxxxx” appeared before and after the sentence with identical speed and size (see Fig. 1). Participants read aloud as many words as possible. The response was self-timed. Corrections were permitted but participants were required to commit to one answer per presented word. The Gazepoint GP3 eye tracker (www.gazept.com) was used to track fixation. Any trials with eye movements impinging more than 2 degrees toward the presented word were repeated using a new sentence.

All sentences were selected randomly from a pool of 2627 sentences extracted from 9 classic novels written in English. The total length of all the sentences used ranged between 40 and 80 characters including spaces.^37^ The average sentence within the pool contained 11 words (SD = 1.7 words). No individual sentence was seen more than once by any participant.

#### Transcranial Electrical Stimulation

Participants underwent one active tES session and one placebo tES session. The order of sessions was randomly selected and counterbalanced. Neither the researcher nor the participant was informed of the order of sessions. The active session was either tDCS (2 mA) or hf-tRNS (2 mA peak to peak). The hf-tRNS current intensity was bounded between –1mA and +1mA with 0 mean.^32^ Both tES protocols lasted 20 minutes and included 30 seconds of ramp up and ramp down. Placebo stimulation involved only the 30 seconds of current ramp up and ramp down, and all other experimental procedures were identical to those used in the active stimulation sessions. The current was delivered with a neuroConn DC Stimulator (Plus or MC, www.neurocaregroup.com) using two 5 × 5 cm rubber electrodes placed inside saline-soaked sponges that were attached to the head with elastic bands.

For participants assigned to the hf-tRNS condition, both electrodes were placed on either side of Oz lengthwise.^28^ The electrodes were placed two to three finger widths apart, and care was taken to maintain a dry section of scalp between electrodes. For participants assigned to the tDCS condition, the anode was placed over Oz using the International 10-20 electroencephalogram (EEG) system. The cathode was placed over a randomly selected cheek which remained consistent across the tDCS active and placebo sessions.^22^^,^^24^

### Experimental Procedure

Participants performed three experimental sessions. In the first session, a threshold reading speed and text size was found without tES. In the second and third sessions, the main experimental task was performed using the thresholds found during the first session.

#### Session 1: Thresholding and parameter selection

The purpose of the thresholding session was to find an appropriate text size and speed for use during the tES sessions that would elicit 55% performance, representing a challenging threshold far from ceiling and floor performance. During thresholding, participants performed the RSVP reading task with five initial text sizes, each with five initial presentation speeds (Table 1). These values were selected from prior pilot testing. Four trials per condition were run, totaling 100 initial trials. Performance accuracy was calculated as the proportion of words identified correctly across all trials exhibiting the same combination of text size and presentation speed. The text sizes were block randomized, and the presentation speeds were randomly interleaved within blocks.

**Table 1.** Initial Text Sizes and Presentation Speeds Presented to Each Participant During Session 1 Thresholding

			Print Size, logMAR		
	0.92	1.08	1.23	1.36	1.43
Presentation	83	50	50	50	50
speeds, ms	150	100	100	100	100
	300	250	200	150	133
	700	450	400	250	233
	2500	1000	1000	800	700

logMAR, logarithm of the minimum angle of resolution; ms, millisecond.

**Figure 2.**
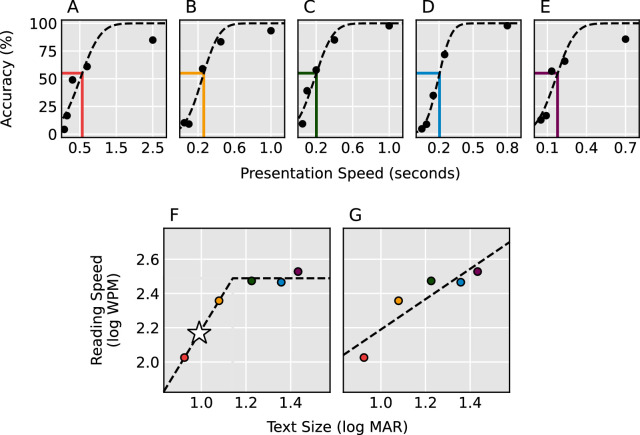
Baseline thresholding procedure for one example participant. Participants performed the RSVP task with five different text sizes: logMAR, (**A**) 0.92, (**B**) 1.08, (**C**) 1.23, (**D**) 1.36, (**E**) 1.43 and a cumulative gaussian psychometric function was fit to the data from each size. The presentation speeds eliciting 55% accuracy for each text size were fit to a continuous piecewise function with one increasing segment and one flat segment (**F**) and a straight line (**G**). Additional print sizes were run until the piecewise function fit the data better than a straight line. The text size and reading speed used during the stimulation sessions were selected by taking the point along the increasing segment corresponding to 0.15 logMAR below the intersection of both segments (*star*).

The presentation speeds were selected to elicit performance spanning 20% to 80% accuracy. Directly after completing the initial 100 trials, the data from each text size were fit to separate cumulative gaussian psychometric functions describing accuracy with changing presentation speed (see Figs. 2A–E for representative psychometric fits). If performance on any text size failed to span the required 20% to 80% performance accuracy, then additional trials were run with new reading speeds to span the full psychometric function.

Subsequently, tested print sizes (logMAR) and their associated presentation speeds (log_10_ words per minute) eliciting 55% accuracy were fit to a linear function and a continuous bilinear piecewise function.^37^ The piecewise function contained one segment that rose with increasing print size up to a maximum “critical print size” (CPS) and one horizontal segment beginning at the CPS and extending to all larger print sizes. The CPS and associated presentation speed demarcates the smallest text with which the participant can achieve their maximum reading speed when targeting a specific accuracy (55% in the current study). The sum of the squared residuals of both models were calculated to verify a reliable CPS. If the linear fit explained equal or greater amounts of the variance compared to the piecewise fit, then additional print sizes were tested to better capture the CPS. This procedure occurred iteratively until the piecewise fit explained more of the variance. See Figures 2F and 2G for representative linear and piecewise fits.

Session 1 was completed after the CPS was found. The print size and presentation speed along the estimated piecewise function 0.15 logMAR below the CPS were selected for use during the participant's tES sessions. The selected parameters fall along the sloped portion of the piecewise function and are therefore theoretically sensitive to improvements in both visual acuity and reading speed.

#### Sessions 2 and 3: tES Experiments

Sessions 2 and 3 were identical, except that one session involved active tES and the other placebo tES in a random order. The experimenters and participants were both blinded to the order of stimulation. Sessions 2 and 3 were separated by at least 2 days and no more than 7 days. On each session, four separate RSVP reading tests were administered. Each test comprised 30 sentences, and performance accuracy was calculated as the total proportion of words identified correctly. The presentation speed and text size were determined by session 1’s parameter selection procedure and remained constant throughout both stimulation sessions. All other RSVP procedures were identical to session 1.

Prior to tES, participants performed an RSVP pre-test. After the pre-test was concluded, either active or placebo tES was applied (hf-tRNS applied bilaterally to early visual areas, tDCS applied with the anode over Oz and the cathode over a randomly selected cheek). A second RSVP test was administered during tES stimulation (“during”). A third test was administered 5 minutes after the stimulation completed (“post5”). The final test was administered 30 minutes after the stimulation completed (“post30”).

## Results

To calculate the effects of active and placebo tES, the pre-test accuracy was subtracted from each of the post-test accuracies. A 2 (Stimulation: active and placebo) × 3 (Time: during, post5, post30) × 2 (Neuromodulation Protocol: tDCS and hf-tRNS) ANOVA was conducted on the difference-from-baseline data. Time and Stimulation were within-subject factors and Neuromodulation Protocol was a between-subjects factor. All analyses were carried out using jamovi software version 2.3 (www.jamovi.org). The full data are presented in Table 2 (hf-tRNS) and Table 3 (tDCS).

**Table 2.** Individual Subject Data From Stimulation Sessions, hf-tRNS

	Active Session Accuracy, %				Placebo Session Accuracy, %			
Subject ID	Pre-Test	During	Post5	Post30	Pre-Test	During	Post5	Post30
1	88.509	96.319	94.277	96.262	92.308	96.979	90.663	93.155
2	82.301	82.006	76.647	81.613	80.236	77.448	80.363	78.698
3	81.194	82.515	87.234	86.293	74.474	69.725	71.646	80.793
4	62.222	66.355	67.456	67.925	58.333	59.633	58.675	59.706
5	79.938	83.596	82.769	88.589	80.380	78.710	83.046	81.515
6	53.659	60.000	50.610	50.588	57.270	55.063	51.622	47.041
7	83.988	88.889	84.840	84.063	79.412	71.845	83.025	81.818
8	87.730	89.877	81.013	87.647	82.298	76.488	79.217	85.152
9	52.322	69.085	76.506	75.504	55.988	66.463	66.361	69.578
10	84.277	90.794	94.540	89.736	92.424	93.548	90.634	95.975
11	81.602	82.477	82.249	84.071	80.000	76.435	81.288	83.125
12	76.301	78.743	73.065	80.403	70.427	69.492	63.988	67.398
13	80.000	79.341	73.148	77.448	78.248	75.510	76.786	76.638
14	70.588	54.192	68.750	64.935	64.286	59.517	63.554	66.768
15	86.068	91.541	88.957	91.738	80.781	87.195	88.060	90.634
16	70.997	74.850	82.883	86.186	82.440	83.686	82.866	86.280
17	85.627	91.279	89.728	88.991	73.795	82.019	82.927	84.524
18	86.068	88.427	90.598	92.025	78.182	78.797	84.026	84.857
19	73.636	80.495	83.235	78.614	78.378	80.531	83.881	80.769
20*	63.988	63.125	67.251	74.224	76.398	40.705	46.688	54.598

hf-tRNS, transcranial random noise stimulation.

% = percent correct.

Pre-Test = Reading test performed before stimulation is administered.

During = Reading test performed while stimulation is administered.

Post5 = Reading test performed 5 minutes after stimulation completed.

Post30 = Reading test performed 30 minutes after stimulation completed.

* Consistent outlier as defined by a 1.5 × IQR threshold. Excluded from analysis.

**Table 3.** Individual Subject Data From Stimulation Sessions, tDCS

	Active Session Accuracy, %				Placebo Session Accuracy, %			
Subject ID	Pre-Test	During	Post5	Post30	Pre-Test	During	Post5	Post30
1	88.462	84.663	88.000	87.798	77.469	84.259	87.147	89.645
2	69.632	79.940	83.284	76.923	79.646	78.963	81.763	80.712
3	77.982	85.531	78.419	72.508	73.125	74.924	75.150	80.982
4	72.136	78.299	68.339	75.776	75.309	76.615	79.487	77.961
5	79.394	90.120	84.706	85.000	88.462	88.991	92.049	85.119
6	50.610	60.790	62.162	59.292	49.394	55.272	66.026	67.069
7	69.394	68.647	75.802	77.885	77.713	79.878	83.232	78.354
8	75.831	68.025	77.551	78.550	82.440	83.792	88.462	89.408
9	76.879	84.639	NA	84.592	75.399	78.743	82.059	81.288
10	74.760	73.065	80.128	82.301	65.846	68.038	72.136	76.115
11	79.814	78.979	76.506	79.701	74.096	83.235	77.204	77.313
12	60.182	58.944	64.565	55.486	53.374	61.667	56.667	64.465
13	78.378	82.934	76.488	78.834	72.012	68.553	68.263	76.602
14	61.538	61.631	68.421	69.322	56.250	66.181	65.282	75.595
15	72.948	70.909	77.326	79.460	67.953	76.667	74.613	71.975
16	83.030	83.284	81.682	84.146	83.129	82.317	82.059	81.763
17	88.485	84.483	83.385	85.801	85.970	82.317	84.375	82.175
18	73.563	73.353	69.162	74.481	72.086	72.673	72.644	77.743
19	78.287	83.766	86.391	88.988	86.880	84.320	89.254	89.728
20	76.667	78.882	79.439	80.303	64.762	67.771	76.563	74.924
21	71.084	68.563	76.945	81.493	78.507	82.749	83.333	83.180

tDCS, transcranial direct current stimulation.

% = percent correct.

Pre-Test = Reading test performed before stimulation is administered.

During = Reading test performed while stimulation is administered.

Post5 = Reading test performed 5 minutes after stimulation completed.

Post30 = Reading test performed 30 minutes after stimulation completed.

NA, data not available.

One hf-tRNS participant exceeded 1.5 × the interquartile range in the baseline-subtracted effect of stimulation and was omitted from the statistical analysis. This anomalous result was driven by poor accuracy during the placebo post-tests relative to pre-test (see Table 2, subject 20).

**Figure 3.**
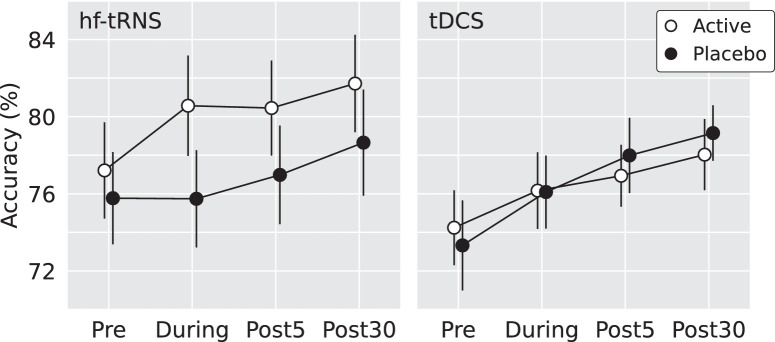
Average performance accuracies for the hf-tRNS and tDCS groups. Error bars are ±1 standard error of the mean.

Mauchly's test found significant deviations of sphericity in Time, χ^2^(2) = 9.5, *P* = 0.009. Therefore, the Greenhouse-Geisser correction (ε = 0.8) was used when evaluating all results involving the Time factor. The main effect of Time was significant, *F*(1.6, 60) = 8.4, *P* = 0.001, but no associated interactions were significant, suggesting a general learning effect (Time * Neuromodulation Protocol: *P* = 0.65, Time * Stimulation: *P* = 0.39, Time * Neuromodulation Protocol * Stimulation: *P* = 0.93). Figure 3 demonstrates clear patterns of within-session learning across all experimental conditions.

**Figure 4.**
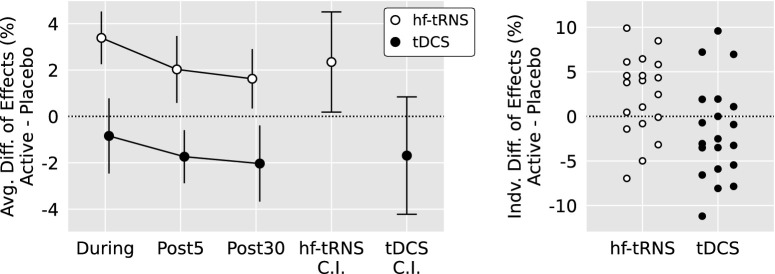
The differences between the effects of active and placebo stimulation for the hf-tRNS and tDCS conditions. *Left plot*: Baseline-subtracted data across each post-test and 95% confidence intervals for the overall difference between active and placebo stimulation. *Right plot*: Overall differences plotted separately for each participant.

There was a significant interaction between Stimulation and Neuromodulation Protocol, *F*(1, 37) = 6.4, *P* = 0.016. Therefore, the effect of Stimulation was assessed separately in participants who had received hf-tRNS and tDCS. Participants who received tDCS did not perform any differently after active stimulation relative to placebo, *P* = 0.18. However, participants who received hf-tRNS significantly improved after active stimulation relative to placebo *F*(1, 18) = 5.2, *P* = 0.035, mean = 2.4%, confidence interval (CI) = 0.18–4.51. Single-subject and overall effects of hf-tRNS and tDCS are plotted in Figure 4.

## Discussion

Active bilateral hf-tRNS to the visual cortex produced a small but significant improvement in peripheral reading, but anodal tDCS to Oz did not produce any benefit. Because the average sentence was approximately 11 words, the overall 2.4% performance improvement induced by tRNS corresponded to roughly 8 additional words read correctly per 30-sentence post-test. It is worth noting that, although the relevant interaction was not significant, the largest numerical improvement occurred while hf-tRNS was actively being administered (that is, the “during” post-test).

The current study found no effect of tDCS. However, Silva et al.^22^ utilized a similar RSVP protocol and reported an effect with tDCS. One of the most pronounced differences between the two studies is how visual fixation was controlled. The current study presented stimuli 10 degrees away from fixation. This relatively large eccentricity was selected because it falls beyond the macula^38^ and has been used in previous work.^39^^,^^40^ In contrast, Silva et al.’s^22^ participants with macular degeneration adopted their choice of fixation eccentricity, potentially the nearest eccentric position possible. Because more eccentric retinotopic positions are located deeper within the calcarine sulcus,^41^ the required stimulation depth likely differed between the current study and the study by Silva et al.^22^

The reason for a null tDCS effect in the current study is unclear, although several possible explanations can be examined in future research. The effects of tDCS are impacted by cortical folding such that protocols designed to impart excitatory stimulation may in fact induce antagonistic excitatory and inhibitory effects on the stimulated area due to the underlying tissue structure.^29^ In contrast, tRNS induces overall excitatory stimulation that is more robust to cortical folding,^29^ potentially enabling more consistent stimulation of the brain region supporting visual processing at 10 degrees eccentricity. In addition, the differential effects of tDCS and hf-TRNS reported here may have been driven partially by differences in electrode placement. Both stimulating electrodes were positioned over the visual cortex during hf-tRNS, allowing both electrodes to contribute to the neuromodulatory effect. However, only the anodal electrode was positioned over the visual cortex during tDCS to minimize the inhibitory effects of cathodal stimulation. Future investigations of the relationship between RSVP reading eccentricity and the efficacy of different tES protocols and electrode montages will be particularly relevant for evaluating potential clinical efficacy. Because visual processing at smaller eccentricities (e.g. 5 degrees) engages brain regions that are closer to the cortical surface and therefore easier to target, it may be especially interesting to determine whether tES produces more consistent improvements in reading at smaller eccentricities.

Although both tDCS and tRNS are implicated in the reduction of visual crowding,^15^ it is also possible that the peripheral reading improvement induced by tRNS was associated with stochastic resonance effects.^32^^,^^33^ Visual acuity is markedly worse at 10 degrees eccentricity than at central fixation, particularly when viewing written words.^42^ High frequency tRNS-induced random noise applied to the early visual areas may have boosted weak peripheral signals, increasing task performance independent of visual crowding. However, whereas visual acuity is poor at 10 degrees eccentricity, our presented stimulus remained visibly suprathreshold for detection. Stochastic resonance is typically understood to act on signals just below the detection threshold and therefore may not account for our reported effect. Because the current study did not explicitly measure crowding, the precise mechanism underlying the observed effect of brain stimulation remains unclear.

The current study used an RSVP paradigm, individually presenting each word to participants and minimizing the influence of eye movements. Participants were not asked to comprehend the sentences. Rather, they performed a visual word recognition task that is presumably less dependent on higher level cognitive processing than sentence comprehension. By presenting this behavioral task concurrently with brain stimulation of early visual cortex via Oz, we aimed to investigate how stimulation affects lower-level visual processing of crowded stimuli. However, just as visual crowding involves a complex network with substantial higher-level contributions,^11^^–^^14^ reading is also a complex cognitive behavior that involves eye movement networks and visual processing at all levels of processing.^43^^–^^45^ Beyond early visual cortex, additional cortical targets exist that may be more directly involved in reading. Therefore, it may be interesting to examine whether larger or more consistent effects will result from tES to higher-level parietal or temporal regions.

Because visual word recognition is dramatically impaired in the periphery,^42^ it was necessary to use individualized presentation speeds and text sizes to control task difficulty across all participants. Whereas our initial baseline thresholding procedure avoided ceiling and floor effects, a clear learning effect between the initial thresholding session and the experimental tES sessions was observed across most participants. We targeted a point below the 55% CPS and corresponding reading speed to enable detection of improvements in either visual acuity or reading speed during the experimental tests. Unfortunately, the elevated pre-tests (see Tables 2, 3) indicate that we cannot be sure where the stimulus fell along each participant's curve describing reading speed as a function of print size, demonstrated in Figure 2F. In addition, consistently stimulating the same brain region across multiple participants is inherently challenging, particularly when targeting a region that is buried deep within a sulcus. Individual variability in our study (see Fig. 4) may have been influenced not only by the chosen peripheral fixation, but also by differences in the brain and skull anatomy of the recruited participants. Because we used a fixed approach to electrode placement, the degree to which relevant brain regions were stimulated likely differed across participants due to natural variations in cortical folding and skull thickness. More consistent effects may be achieved in future work by better controlling task difficulty and using electric field modeling based on individual anatomic structure to guide electrode placement.^46^^,^^47^

Finally, we found a general within-session learning effect during all sessions. A similar within-session learning effect was recently found in a study examining the effect of tDCS on peripheral reading of Chinese characters.^48^ Enhancement of peripheral reading with repeated practice is well-established in participants with normal vision and in participants with visual deficits.^39^^,^^49^^–^^53^ Although these perceptual learning paradigms improve peripheral reading, they require multiple sessions and adherence can be difficult. TES can induce neuroplastic changes that result in improved sensitivity to visual stimuli.^54^^,^^55^ More recent work has demonstrated that brain stimulation can improve perceptual learning in participants with macular degeneration performing non-reading tasks.^20^ Future work will examine whether augmenting training with tES may promote faster and more effective reading improvement than either methodology alone.

## Conclusions

Noninvasive brain stimulation of early visual areas may be potentially useful for improving the visual processing of text presented to the periphery. Although the effect reported here is statistically significant, it is small, with an average accuracy improvement of 2.4%. Nevertheless, this study raises questions about the dependence of the effect of brain stimulation on various experimental factors, including viewing eccentricity, task difficulty, electrode montage, and stimulation protocol. Additional work is required to better clarify the conditions that promote the most reliable effects across all participants. In addition, our results are consistent with previous findings that repeated testing and training of peripheral reading improves performance.
